# Gut dysbiosis: Ecological causes and causative effects on human disease

**DOI:** 10.1073/pnas.2316579120

**Published:** 2023-12-04

**Authors:** Sebastian E. Winter, Andreas J. Bäumler

**Affiliations:** ^a^Department of Medicine, Division of Infectious Diseases, University of California, Davis, CA 95616; ^b^Department of Medical Microbiology and Immunology, University of California, Davis, CA 95616

**Keywords:** microbiota, dysbiosis, colitis, colorectal cancer

## Abstract

The gut microbiota plays a role in many human diseases, but high-throughput sequence analysis does not provide a straightforward path for defining healthy microbial communities. Therefore, understanding mechanisms that drive compositional changes during disease (gut dysbiosis) continues to be a central goal in microbiome research. Insights from the microbial pathogenesis field show that an ecological cause for gut dysbiosis is an increased availability of host-derived respiratory electron acceptors, which are dominant drivers of microbial community composition. Similar changes in the host environment also drive gut dysbiosis in several chronic human illnesses, and a better understanding of the underlying mechanisms informs approaches to causatively link compositional changes in the gut microbiota to an exacerbation of symptoms. The emerging picture suggests that homeostasis is maintained by host functions that control the availability of resources governing microbial growth. Defining dysbiosis as a weakening of these host functions directs attention to the underlying cause and identifies potential targets for therapeutic intervention.

## Significance

Few recent advances in human medicine have been as influential as the finding that an imbalance (dysbiosis) of our resident microbial communities in the colon is linked to many chronic human illnesses. However, translating advances in microbiome research into clinical interventions requires a better understanding of the ecological causes of dysbiosis and the causative effects dysbiosis has on human disease. Recent progress in answering these questions suggests that the host determines which redox reactions are available for microbial growth by controlling the availability of respiratory electron acceptors. Dysbiosis is characterized by an increased availability of host-derived electron acceptors, which changes microbiota composition and function. These insights provide an alternative starting point for approaches to rebalance the microbiota.

The human colon harbors a microbial community (microbiota) that is 100-fold larger than any other bacterial consortium inhabiting our body (1). Its magnitude makes the colonic microbiota a major source of microbial metabolites that influence human physiology (2–5). Fecal microbiota profiling reveals that a shift in the species composition, termed gut dysbiosis, is observed in diseases that include leading causes of human morbidity and mortality, such as cardiovascular disease (CVD) (6), diabetes (7, 8), colorectal cancer (CRC) (9), chronic kidney disease (CKD) (10), and inflammatory bowel disease (IBD) (11). These observations raise the intriguing prospect that the colonic microbiota plays a central role in human health (12).

Here, we examine the obstacles encountered in understanding microbiome homeostasis through analysis of the microbiota and their genes. We will then discuss how recent advances in the field of bacterial pathogenesis research delivered seminal insights into the ecological causes of dysbiosis, which are directly applicable to microbiome research. Finally, we will consider how understanding the ecological causes of dysbiosis opens avenues for establishing causative links to human disease and mitigating its adverse effects.

## Omics, Omics, Everywhere, Nor Any Healthy Microbiome Link

Modern microbiome research is rooted in the innovation of culture-independent methods for profiling complex microbial communities, an approach powered by advances in high-throughput sequencing. Initially, this technology generated catalogs of microbial species names, which focused early work on the question of what a normal species composition of the human gut microbiota looks like. The first step in defining a “healthy” microbial community is the identification of core species that are common to the colonic microbiota of humans (13). However, determining abundant core species that define a healthy human fecal microbiota turned out to be challenging since this microbial community exhibits marked interpersonal differences in bacterial species content (14, 15). The finding that each person’s gut microbial community varies in the specific bacterial species present does not support the hypothesis that there is a healthy core gut microbiota defined by a set of microbial species that we all share (15).

The absence of core species in the human colonic microbiota makes it difficult to define gut homeostasis by profiling the microbiota composition. This impasse led some to suggest that a healthy human microbiome might not even exist (16). Dysbiosis is commonly defined as a decrease in microbial diversity, an absence of beneficial microbes or the presence of potentially harmful microorganisms (17). However, our inability to identify abundant core species that define a healthy human microbiome challenges the idea that gut dysbiosis can be defined based on changes in the species composition. Proponents of this standpoint go as far as to suggest that the term dysbiosis is a distraction from useful microbiome research (18). These limitations and controversies illustrate why microbiota profiling alone does not provide a straightforward path toward understanding the ecological causes of dysbiosis.

Although a healthy human gut microbiota cannot be defined by a set of core species, a core gut microbiome does exist at the level of physiological, often metabolic, functions. Metagenomic analysis of the human fecal microbiota reveals that despite interpersonal variation in species assemblages, there is an identifiable core of genes encoding various metabolic pathways, such as those involved in carbohydrate and amino acid metabolism (15). However, it is difficult to draw clear connections between metagenomic data and the health of a particular microbiome. These limitations persist although technology continues to advance as metatranscriptomics and metabolomics replace metagenomics. At the completion of the Human Microbiome Project in 2019 (19), a commentary summarized the state of the human microbiome field by remarking that despite generating 42 terabytes of multiomics data, “researchers don’t yet agree what constitutes a healthy microbiome or how to define an impaired one” (20). The inability to define a healthy human microbiota despite a wealth of high-throughput sequencing data evokes parallels to the notorious phrase from Samuel T. Coleridge’s Rime of the Ancient Mariner, “water, water, everywhere, nor any drop to drink*”.*

The question remains why cataloging bacterial species, their genes, and gene products does not provide a straightforward path for translating vast amounts of data into a better understanding of microbiome health. Since the microbiome is commonly defined as the collection of all microbes and their genes (21), it seems at first puzzling that a massive amount of data covering every aspect of the microbiome has not resulted in a better understanding of what it looks like during health. However, ecological theory suggests that microbes and their genes are only one part of the microbiome, which is defined ecologically as the microbiota and its environment, including the body part inhabited by the microbial community (22, 23). This alternative viewpoint suggests that microbiota profiling, metagenomics, and metatranscriptomics provide an incomplete picture of the microbiome since no direct measurements of the host environment are included in the analysis (24). As discussed below, incorporating the host environment into microbiome analysis does indeed provide the missing information to unravel the ecological causes of dysbiosis because this condition commonly features an increase in host-derived resources required for respiration, the most efficient driver of microbial growth. However, to review the origins of this conceptual advance, we must switch to the field of infectious disease research.

## Pathogens Expose Causative Links between Host Physiology and Gut Microbiota Composition

### Pathogen-Induced Dysbiosis.

During homeostasis, the colonic microbiota is dominated by bacteria belonging to the classes *Clostridia* (phylum *Bacillota*) and *Bacteroidia* (phylum *Bacteroidota*) (25). However, intestinal inflammation triggered by virulence factors of enteric pathogens, such as *Salmonella enterica* serovar (*S.*) Typhimurium (class *Gammaproteobacteria*; phylum *Pseudomonadota*), *Citrobacter rodentium* (class *Gammaproteobacteria*), or the parasite *Toxoplasma gondii* (phylum *Apicomplexa*), drives dysbiosis characterized by an increase in the abundance of bacteria belonging to the classes *Gammaproteobacteria* and *Bacilli* (phylum *Bacillota*) (26–28). The pioneering studies describing this phenomenon kicked off a new line of research seeking to understand how pathogen-indued inflammation changes the microbiota composition (29).

### Tetrathionate.

**Fig. 1.**
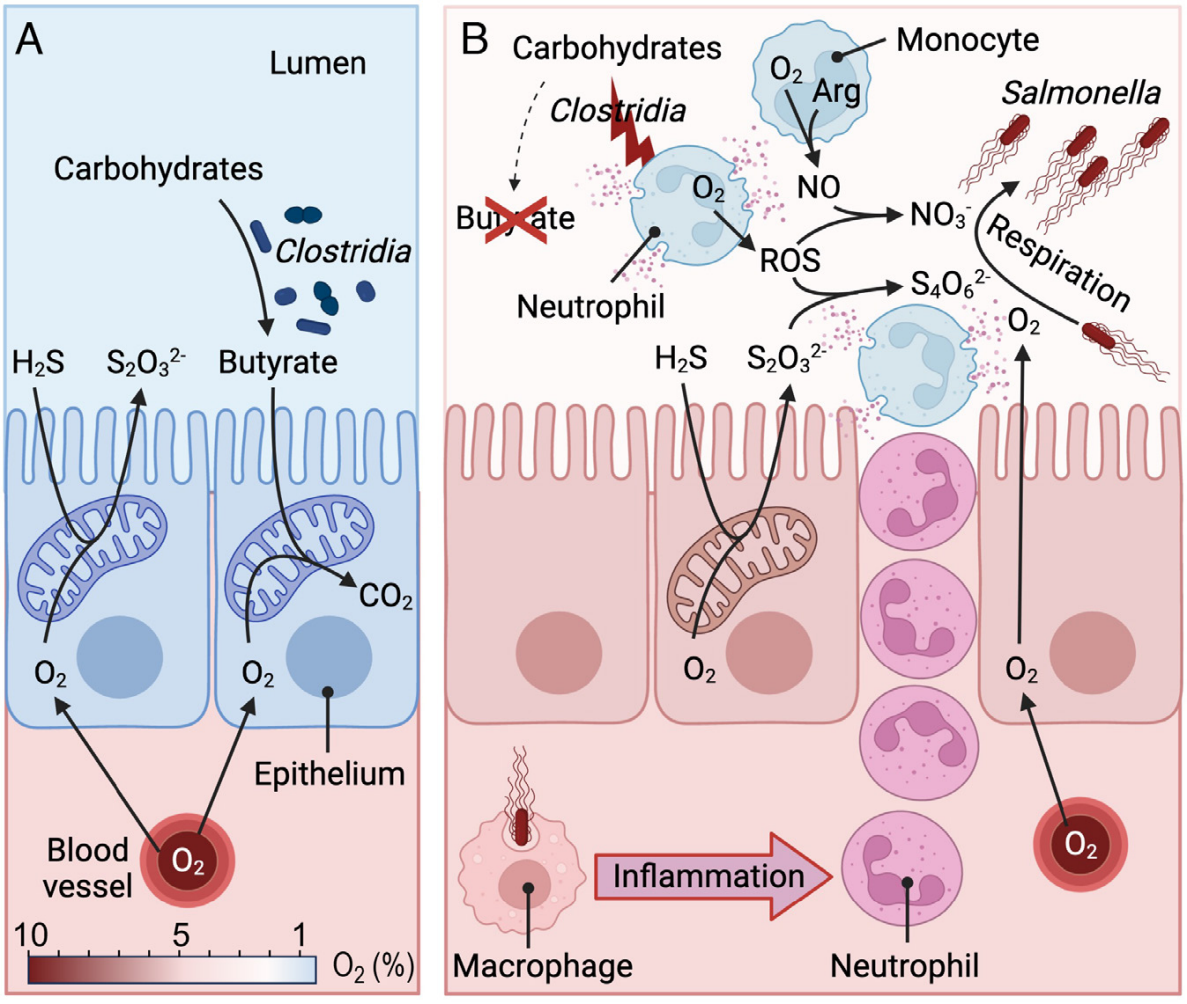
Pathogen-induced colitis increases the availability of host-derived respiratory electron acceptors to increase pathogen abundance in the colon. (*A*) During homeostasis, oxygen (O_2_)-consuming reactions maintain the colonic epithelium in a state of physiological hypoxia. (*B*) During *Salmonella* infection, invasion of the intestinal mucosa by the pathogen triggers transepithelial migration of neutrophils, resulting in a depletion of *Clostridia* and loss of epithelial hypoxia. NO, nitric oxide; ROS, reactive oxygen species; NO_3_^−^; nitrate; S_2_O_3_^2−^, thiosulfate; H_2_S, hydrogen sulfide, S_4_O_6_^2−^, tetrathionate; CO_2_, carbon dioxide; Arg, arginine. Created with BioRender.com.

One beneficiary of gut inflammation is *Salmonella Typhimurium* because colitis triggered by the invasive pathogen promotes fecal–oral transmission by increasing its abundance in the feces (30). A breakthrough in understanding how colitis boosts growth of *S. Typhimurium* was the finding that phagocytes recruited into the intestinal lumen during gut inflammation provide a respiratory electron acceptor for the pathogen by oxidizing endogenous sulfur compounds to tetrathionate (S_4_O_6_^2−^) (31) (Fig. 1). Tetrathionate has been used for the past century by clinical laboratories to enrich for *S. Typhimurium* in samples containing competing microbes (32). But in the colon, the use of tetrathionate as an electron acceptor for anaerobic respiration gives the pathogen an edge over bacteria that rely on fermentation for growth. Importantly, a combination of bacterial and host genetics made it possible to establish a causative link between the generation of host-derived tetrathionate during colitis and enhanced pathogen growth within the gut microbiota (31).

### Nitrate.

The finding that the host provides tetrathionate to boost growth of *S. Typhimurium* was soon followed by the finding that additional respiratory electron acceptors are generated during pathogen-induced colitis. Phagocytes recruited into the intestinal lumen during inflammation release superoxide (O_2_^−^) and nitric oxide radicals (NO) produced by phagocyte Nicotinamide Adenine Dinucleotide Phosphate (NADPH) oxidase (33) and inducible nitric oxide synthase (iNOS) (34), respectively. Superoxide and nitric oxide react to form peroxynitrite (ONOO^−^) (35, 36), an unstable bactericidal compound that decomposes to nitrate (NO_3_^−^) (37) (Fig. 1). Thus, a by-product of recruiting phagocytes into the intestinal lumen during *S. Typhimurium* infection is an elevated concentration of host-derived nitrate in the mucus layer (38, 39). Nitrate is used by *S. Typhimurium* as an electron acceptor for anaerobic respiration (40). By powering nitrate respiration, intestinal inflammation fuels growth of *S. Typhimurium* to increase the pathogen abundance in the feces (38, 39).

Host-derived nitrate is also generated when phagocytes migrate into the intestinal lumen in response to infection with the parasite *Toxoplasma gondii* (41). By increasing the nitrate concentration in the lumen of the small intestine, *T. gondii*–induced inflammation drives dysbiosis characterized by an elevated abundance of commensal *Escherichia coli* (class *Gammaproteobacteria*) because this commensal can accelerate its growth through anaerobic nitrate respiration (41). These data suggest that host-derived nitrate generated as a by-product of intestinal inflammation is an ecological driver of changes in the composition of gut-associated microbial communities during inflammation (42).

### Oxygen.

*S. Typhimurium*–induced colitis results in a loss of anaerobiosis by increasing the diffusion of host-derived oxygen into the lumen. This process is initiated by luminal phagocytes that release antimicrobials during *S. Typhimurium*–induced colitis, thereby lowering the microbial density (43, 44) and altering the composition of the gut microbiota by reducing the abundance of *Lachnospiraceae* (class Clostridia) and *Ruminococcaceae* (class Clostridia) (45). *Lachnospiraceae* and *Ruminococcaceae* are the main producers of the short-chain fatty acid (SCFA) butyrate within the gut microbiota (46, 47). A reduced abundance of these butyrate-producing taxa during *S. Typhimurium* infection leads to a marked drop in the luminal butyrate concentration (48). Butyrate is an important signal regulating energy metabolism in colonic epithelial cells (49, 50). During gut homeostasis, butyrate-signaling preserves high mitochondrial oxygen (O_2_) consumption through oxidative phosphorylation, thereby maintaining the colonic surface in a state of physiological hypoxia (<1% O_2_) compared to normal levels of tissue oxygenation (3 to 10% O_2_) (51, 52) (Fig. 1*A*). Physiological hypoxia of the colonic epithelium limits diffusion of oxygen into the intestinal lumen to maintain anaerobiosis (53). Depletion of butyrate causes colonic epithelial cells to reduce mitochondrial oxygen consumption and shift their energy production toward a conversion of glucose into lactate (aerobic glycolysis), which increases epithelial oxygenation (51). This heightens the amount of oxygen emanating from the epithelial surface, thereby driving growth of *S. Typhimurium* in the gut lumen through aerobic respiration (48) (Fig. 1*B*).

Likewise, a disruption of anaerobiosis is observed during infection with the luminal pathogen *C. rodentium*, which increases epithelial oxygenation through a different mechanism than *S. Typhimurium* (54). *C. rodentium* uses its virulence factors to attach to and damage epithelial cells in the large intestine, which triggers an epithelial repair response (55). Epithelial cell numbers are increased by excessive cell division of undifferentiated epithelial cells, termed transit-amplifying (TA) cells, which expand from their usual location in the crypts to reach the epithelial surface (54). This regenerative response leads to a longitudinal increase in epithelial cell numbers in the crypts, which is visible as crypt elongation, a lesion known as colonic crypt hyperplasia (56, 57). Unlike differentiated epithelial cells that consume oxygen through oxidative phosphorylation in their mitochondria (49), TA cells obtain energy through aerobic glycolysis(58) and therefore do not exhibit hypoxia. By replacing differentiated epithelial cells with TA cells on the mucosal surface, *C. rodentium*–induced colonic crypt hyperplasia increases epithelial oxygenation, and the resulting surge of oxygen diffusing into the intestinal lumen fuels pathogen growth through aerobic respiration (54).

### Fermentation vs. Respiration.

The discovery that infectious colitis increases the availability of exogenous respiratory electron acceptors explains an increase in the luminal abundance of enteric pathogens (31, 38, 48, 54), but the broader implications are that these changes in the host environment are an ecological driver of gut dysbiosis (41). To appreciate the role host-derived electron acceptors play in changing the composition of microbial communities, it is helpful to reflect on how their availability governs the abundance of bacteria that rely on fermentation for growth, i.e., obligately anaerobic bacteria, vs. bacteria that can support their grow by respiring oxygen or nitrate, i.e., facultatively anaerobic bacteria.

One basic tenet of microbial population biology is that taxa abundance is a function of the bacterial growth rate, as species with the shortest generation time come to dominate microbial communities. The bacterial growth rate is limited by energy metabolism, in particular the availability of adenosine triphosphate (ATP) (59). ATP is generated through redox reactions, in which the transfer of electrons from an electron donor, such as glucose, to an electron acceptor, such as oxygen, is coupled to oxidative phosphorylation or substrate-level phosphorylation. In general, the amount of ATP that can be generated from an electron donor, such as glucose, increases with the redox potential (E’_0_) of the electron acceptor. The redox potential is highest for oxygen (E’_0_ for O_2_/H_2_O = 820 mV), followed by nitrate (E’_0_ for NO_3_^−^/NO_2_^−^ = 433 mV), and lower for endogenous electron acceptors used for fermentation, such as pyruvate (E’_0_ for pyruvate/lactate = −190 mV). As a result of this thermodynamic hierarchy of electron acceptors, the growth yield of *Klebsiella aerogenes* (class *Gammaproteobacteria*) on glucose under anaerobic conditions is almost doubled when nitrate is present and almost tripled when bacteria are cultured aerobically (60). As taxa abundance is a function of the amount of ATP generated through redox reactions, the influence host-derived electron acceptors exert over the microbiota composition is dominant over effects arbitrated by electron donors in the diet. By controlling the availability of host-derived electron acceptors, the host selects which metabolic traits can succeed in the gut environment by managing the top level in the hierarchy of factors determining the microbial growth rate. In practical terms, management of electron acceptor availability provides the host with a mechanism to control the abundance of obligately vs. facultatively anaerobic bacteria.

Since phylogeny is a good predictor of complex metabolic traits (61), a shift from obligately to facultatively anaerobic bacteria results in a class-level change in the gut microbiota composition. The ability to respire oxygen or nitrate is widely conserved among facultatively anaerobic bacteria, such as *Gammaproteobacteria* or *Bacilli*, but largely absent in obligately anaerobic bacteria, including *Clostridia* and *Bacteroidia* (62). Therefore, in the presence of host-derived oxygen or nitrate, *Gammaproteobacteria* and *Bacilli* are predicted to have the fastest growth rate, which explains their increased abundance during pathogen-induced colitis (26–28). Conversely, when oxygen and nitrate are not available, obligately anaerobic bacteria that specialize on fermentation exhibit the fastest growth rate, which drives the observed dominance of *Clostridia* and *Bacteroidia* during homeostasis (25).

### Homeostasis vs. Dysbiosis.

Dysbiosis has traditionally been defined in terms of the changes in the bacterial species composition of the gut microbiota (17), but insights into the ecological causes of dysbiosis create a new paradigm, which shifts the focus away from bacterial species and toward understanding the underlying changes in host physiology (24). The host maintains homeostasis by restricting the availability of oxygen and nitrate, thereby regulating the composition of the colonic microbiota by controlling the luminal environment (55, 63, 64). Conversely, dysbiosis involves a change in the host environment characterized by an increased availability of host-derived electron acceptors (24, 63, 65). This environment fuels growth of facultatively anaerobic bacteria to generate a microbial signature of dysbiosis (66) that serves as a biomarker for the underlying alteration in host physiology (67). This paradigm shift suggests that dysbiosis represents a state of weakened host control over the microbial environment, whereas gut homeostasis defines a state where these host functions operate normally (24, 63, 65, 68).

Notably, an elevated availability of host-derived electron acceptors creates a microbial signature of dysbiosis that is not defined by the presence or prevalence of specific core species, but by the relative success of a metabolic trait. Facultatively anaerobic bacteria form a metabolic guild because they use the same resources, i.e., respiratory electron acceptors, in a similar way (69). This metabolic trait might be represented by different bacterial species in different individuals, but an increased abundance of facultatively anaerobic bacteria can be detected even in the face of marked interpersonal differences in bacterial species content.

Importantly, the microbial signature of dysbiosis generated during infection with enteric pathogens (26–28) is also observed in numerous noncommunicable human diseases (66, 67). For example, an increased abundance of facultatively anaerobic bacteria in the fecal microbiota is detected in patients with IBD (11), CRC (9), cancer cachexia (70), radiation enteritis during radiotherapy (71), CKD (10), type 1 diabetes (7), nonalcoholic steatohepatitis (72), graft vs. host disease (73), severe malnutrition (kwashiorkor) (74), chronic inflammation during aging (inflammageing) (75), and CVD (6). Furthermore, this microbial signature of dysbiosis is associated with chronic alcohol consumption (76), and with exposure to a diet that contains high levels of saturated fatty acids (77), two environmental risk factors for developing CVD (78, 79). By generating insights into the mechanistic underpinnings of this microbial signature of dysbiosis, infectious disease research offers a fresh starting point for understanding the ecological drivers of dysbiosis in many noninfectious human conditions.

## Ecological Causes of Dysbiosis

### Ulcerative Colitis.

Mechanistic insights into pathogen-induced dysbiosis have helped open new avenues for research into the causes of dysbiosis during IBD. IBD is an illness characterized by chronic mucosal inflammation of the digestive tract. Genetic and environmental risk factors are thought to trigger IBD by generating inappropriate mucosal immune activation that is driven by the gut microbiota. This consensus view predates modern microbiome research (80), but the underlying mechanisms are still obscure. One form of the disease, termed ulcerative colitis (UC), remains restricted to the colon, where it triggers dysbiosis characterized by an increased *Gammaproteobacteria* and decreased *Clostridia* abundance (81, 82). Insights into the mechanisms that drive dysbiosis during infection with enteric pathogens suggest that an elevated *Gammaproteobacteria* abundance is likely the result of a rise in host-derived respiratory electron acceptors (31, 38, 41, 48, 54, 83). This prediction raises two questions: i) is the electron acceptor hypothesis consistent with clinical observations and ii) does this hypothesis hold up to scrutiny when tested in animal models.

UC patients exhibit increased concentrations of nitric oxide in luminal gas and elevated nitrate levels in their feces (84, 85). This increase in the luminal concentration of nitrate in the large intestine is recapitulated in mouse models of genetically or chemically induced colitis (86). Nitrate generated during colitis is host-derived because its production can be abrogated either by chemical inhibitors of iNOS or by genetic deletion of the murine *Nos2* gene, which encodes iNOS (86). The use of bacterial genetics reveals that increased levels of host-derived nitrate during colitis are causatively linked to an elevated abundance of commensal *E. coli* in the gut microbiota (86). Collectively, these data support the notion that a heightened availability of host-derived nitrate is one of the ecological causes of dysbiosis in UC (42).

**Fig. 2.**
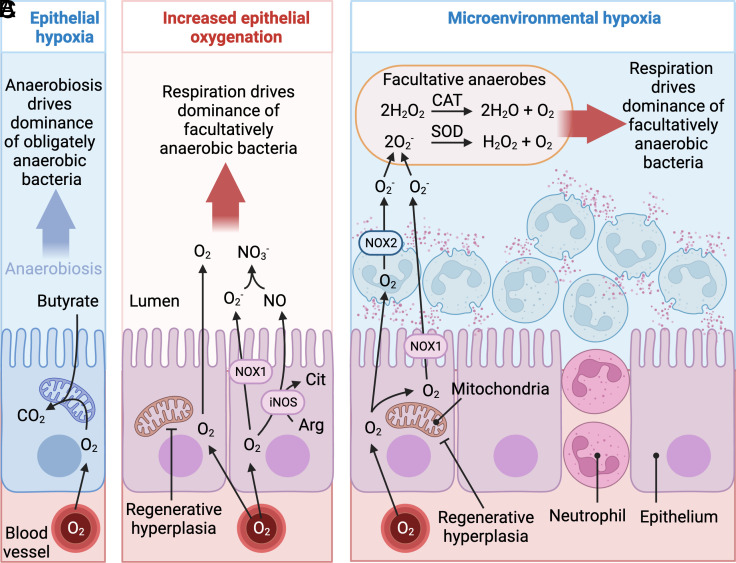
The ecological causes for an intestinal domination of facultatively anaerobic bacteria in the fecal microbiota during UC. (*A*) During homeostasis, mitochondrial oxygen (O_2_) consumption maintains the colonic epithelium in a state of physiological hypoxia. (*B*) Epithelial injury during UC triggers regenerative hyperplasia to increase diffusion of oxygen into the intestinal lumen. (*C*) Neutrophil transepithelial migration during UC leads to depletion of microenvironmental oxygen by the phagocyte NADPH oxidase (NOX2), which generates microenvironmental hypoxia at the mucosal surface. O_2_^−^, superoxide; CO_2_, carbon dioxide; H_2_O_2_, hydrogen peroxide; H_2_O, water; Arg, arginine; Cit, citrulline; NO, nitric oxide; ROS, reactive oxygen species; NO_3_^−^; nitrate; iNOS, inducible nitric oxide synthase, NOX1, epithelial NADPH oxidase, CAT, catalase, SOD, superoxide dismutase. Created with BioRender.com.

Direct measurements of oxygen levels in the colon of UC patients are not available. However, maintenance of epithelial hypoxia requires high mitochondrial oxygen consumption (51) (Fig. 2*A*). Notably, UC patients exhibit reduced mitochondrial function in colonic epithelial cells compared to healthy controls (87–89), which is consistent with the idea that epithelial oxygenation becomes elevated during UC. Experimental support for the oxygen hypothesis comes from a mouse model of dextran sulfate sodium (DSS)-induced colitis. Metagenomic sequencing reveals that metabolic pathways involved in aerobic respiration are overrepresented in the colonic microbiota of mice with DSS-induced colitis (90). Oxygen availability can become elevated during DSS-induced colitis through two mechanisms. The first is a consequence of DSS-induced endoplasmatic reticulum stress in epithelial cells, which results in epithelial injury (91, 92). DSS-induced epithelial injury stimulates regenerative hyperplasia, a process that eliminates physiological hypoxia of the colonic epithelium (93, 94) (Fig. 2*B*). In turn, elevated epithelial oxygenation increases diffusion of oxygen into the lumen to boost growth of commensal *E. coli* by aerobic respiration (90, 93, 94).

The second mechanism offering access to oxygen during DSS-induced colitis is a consequence of mucosal abnormalities that are linked to neutrophil transepithelial migration. During the respiratory burst of neutrophils, the phagocyte NADPH oxidase (NOX2) generates superoxide radicals by rapidly depleting microenvironmental oxygen (O_2_ + NADPH → O_2_^−^ + NADP^+^). This process generates a microenvironmental hypoxia in areas of the mucosal surface that are affected by transmigration of neutrophils (95). Commensal *E. coli* can use superoxide dismutase to disproportionate superoxide radicals to hydrogen peroxide and oxygen (2O_2_^−^ + 2H^+^ → H_2_O_2_ + O_2_), followed by catalase-mediated conversion of hydrogen peroxide to oxygen and water (2H_2_O_2_ → O_2_ + 2H_2_O). Oxygen liberated by these reactions can then be used for aerobic respiration, a process contributing to growth of commensal *E. coli* in the colon of mice with colitis (96) (Fig. 2*C*).

In summary, existing clinical data and the available experimental evidence from animal models point to an increased accessibility of host-derived oxygen and nitrate in the intestinal lumen as a cause for the elevated abundance of facultatively anaerobic bacteria in the feces of UC patients. This body of work suggests that the ecological causes of dysbiosis during UC represent those previously implicated in driving dysbiosis during infection with enteric pathogens.

### Exposure to Antibiotics.

Since inflammation is a stereotypic host response, it is perhaps not surprising that the ecological causes of dysbiosis are similar for infectious colitis and UC. However, an elevated abundance of facultatively anaerobic bacteria in the fecal microbiota is a signature of dysbiosis that is also linked to antibiotic therapy (97, 98), which does not induce colitis.

Clinically, the use of broad-spectrum antibiotics perturbs the normal gut microbiota and sets the stage for intestinal domination by endogenous *Gammaproteobacteria* or *Bacilli* (99). Of special concern are carbapenem-resistant *Enterobacteriaceae* (class *Gammaproteobacteria*) and vancomycin-resistant *Enterococcus faecalis* (class *Bacilli*) because in hematopoietic cell transplantation recipients, an intestinal domination by these antibiotic-resistant opportunistic pathogens is a source of bloodstream infections with high mortality rates (98–100).

An increase in the abundance of *Gammaproteobacteria* and *Bacilli* is recapitulated in mice after oral administrations of antibiotics (101–103). The phenomenon has been studied since the 1950s using streptomycin treatment of mice followed by exogenous administration of commensal or pathogenic *Enterobacteriaceae* (104–110). Early work in this model shows that a perturbation of the microbiota with streptomycin depletes the SCFAs acetate, propionate, and butyrate, which is associated with an increase in the luminal redox potential (110). More recent data unveil that streptomycin treatment results in a loss of epithelial hypoxia (51) and an induction of mucosal *Nos2* expression (111). The mechanism underlying these changes is an antibiotic-mediated depletion of SCFAs, which triggers a metabolic reprogramming of differentiated colonic epithelial cells to increase the availability of oxygen and nitrate (53).

Depletion of SCFAs generates two distinct changes in host physiology that participate in this metabolic reprogramming of epithelial cells. The first is a loss of epithelial signaling through PPAR-γ (peroxisome proliferator-activated receptor gamma), a nuclear receptor that is synthesized by differentiated epithelia cells of the colon (112, 113). During homeostasis, *Clostridia-*derived butyrate activates epithelial PPAR-γ-signaling (114), which results in suppression of iNOS synthesis (115) and activation of mitochondrial bioenergetics (49, 116). By depleting butyrate, streptomycin treatment reduces epithelial PPAR-γ-signaling, resulting in elevated epithelial *Nos2* expression and an increase in the nitrate concentrations in the mucus layer (117).

The second signal required for a metabolic reprogramming of the epithelium during streptomycin treatment is linked to a reduction in the colonic pool of regulatory T cells (T_regs_). Microbiota-derived SCFAs signal through G-protein coupled receptors in host cells to maintain the regulatory T cell pool in the colonic mucosa (118–121). By depleting SCFAs, streptomycin treatment reduces the numbers of T_regs_ in the colonic mucosa (118, 121). A reduction in the numbers of these immunosuppressive cells triggers low-grade mucosal inflammation (111), which generates a type I interferon-dependent signal needed by epithelial cells to undergo metabolic reprogramming (122). Neither a reduction in the numbers of colonic T_regs_, nor a loss of epithelial PPAR-γ-signaling alone are sufficient for eliminating epithelial hypoxia in the colon. But by combining these two changes in host physiology, a streptomycin-mediated depletion of SCFAs reduces mitochondrial oxygen consumption in the epithelium to increase the diffusion of oxygen into the colonic lumen (117).

Collectively, data from the streptomycin-treated mouse model suggest that a depletion of microbiota-derived SCFAs during antibiotic therapy triggers a metabolic reprogramming of colonic epithelial cells that increases the availability of host-derived oxygen and nitrate in the intestinal lumen. Importantly, this increase in the availability of host-derived electron acceptors drives an intestinal domination by commensal *E. coli*, which is causatively linked to their ability to respire oxygen and nitrate (111, 117). Thus, the ecological causes of dysbiosis during antibiotic therapy are explained by some of the same environmental changes that increase the abundance of facultatively anaerobic bacteria during infection-induced colitis.

### A Common Driver of Dysbiosis in Noncommunicable Diseases.

More recent work reveals that a rise in the concentration of host-derived respiratory electron acceptors in the colon is not limited to antibiotic therapy or UC but also explains an elevated fecal abundance of facultatively anaerobic bacteria in several other human diseases. For instance, an altered gut epithelial metabolism and host-derived nitrate boost luminal growth of *Klebsiella oxytoca* (class *Gammaproteobacteria*) in a mouse model of cancer cachexia (70). Similarly, in a mouse model of CRC, an increase in the abundance of *Gammaproteobacteria* can be functionally linked to an impaired ability of the host to limit the availability of oxygen and nitrate during intestinal inflammation (93, 123). Furthermore, an elevated abundance of *E. coli* in the colonic microbiota during high fat intake (77), can be explained by a rise in host-derived oxygen and nitrate in the colon (124). Finally, in a mouse model of graft vs. host disease, loss of physiological hypoxia in the colonic epithelium is linked to dysbiosis characterized by an increased abundance of *Gammaproteobacteria* in the fecal microbiota (125).

The emerging concept that metabolic reprogramming of the colonic epithelium shapes the microbial environment to favor growth of facultatively anaerobic bacteria (53, 65, 67, 126) provides a promising starting point for studying human diseases in which the ecological causes for gut dysbiosis remain unknown. Low-hanging fruits for testing this concept are conditions associated with reduced mitochondrial function, which is indicative of increased epithelial oxygenation (51, 117). For example, mitochondrial dysfunction associated with senescence is a feature of inflammageing (127), but it remains to be tested whether it triggers a loss of epithelial hypoxia in the colon to explain the increased abundance of *Gammaproteobacteria* in the feces of patients (75). Similarly, mitochondrial dysfunction of colonic epithelial cells is observed in a mouse model of type 1 diabetes (128), but its potential impact on epithelial hypoxia and the overgrowth of *Gammaproteobacteria* in the fecal microbiota of patients remain unexplored (7). Finally, consumption of excessive quantities of alcohol triggers mitochondrial abnormalities in the colonic epithelium of patients (129), but it has never been examined whether a loss of epithelial hypoxia is an ecological driver of the increased *Enterobacteriaceae* abundance in the fecal microbiota of patients with alcohol dependence (76).

To summarize, by incorporating the host environment into microbiome analysis, infectious disease research identifies ecological causes of dysbiosis that are germane for understanding the mechanistic underpinnings of gut dysbiosis in a broad spectrum of noncommunicable human diseases. In the following paragraphs, we will consider how this information helps improve our understanding of potential consequences of dysbiosis.

## Causative Effects of Dysbiosis on Disease

### Metabolism-Based Editing of the Microbiota: IBD.

Insights into the ecological causes of dysbiosis suggest that this condition is secondary to an underlying defect in the host that weakens functions involved in controlling the microbial environment (53, 65, 67, 68). However, changes in microbiota composition and function during dysbiosis can contribute to disease. For example, antibiotic therapy can induce remission in IBD, suggesting that the microbiota contributes to intestinal inflammation (130). Similarly, transfer of dysbiotic microbial communities exacerbates intestinal inflammation in mouse models of IBD (131–133).

A prominent microbial signature of dysbiosis in IBD is a bloom of *Enterobacteriaceae* in the fecal microbiota (134). *Enterobacteriaceae* are proinflammatory because their lipopolysaccharide is a potent inducer of innate immune pathways. The proinflammatory nature of *Enterobacteriaceae* might be relevant because IBD is associated with decreased barrier function (135) and increased bacterial translocation to the mesenteric lymph nodes (136). However, to establish that an increased abundance of *Enterobacteriaceae* is causatively linked to a worsening of symptoms, it is necessary to demonstrate that a targeted approach for reducing their abundance (i.e., without changing the prevalence of other taxa) provides symptom relieve.

A possible strategy for normalizing the abundance of *Enterobacteriaceae* during colitis is to selectively block the metabolic pathways that enhance their growth during gut inflammation. Metagenomic sequencing shows that metabolic functions linked to aerobic and anaerobic respiration are overrepresented during DSS-induced colitis in mice (90). In *Enterobacteriaceae*, a subset of terminal reductases involved in these metabolic pathways require insertion of a molybdenum (Mo)-containing cofactor (molybdopterin) into their active sites (137). *E. coli* mutants deficient for molybdopterin biosynthesis can no longer take advantage of the increased availability of respiratory electron acceptors to boost their growth in the murine large intestine during colitis (86). Tungsten (W) can replace Mo in molybdopterin, rendering this cofactor inactive in *Enterobacteriaceae* (138). In mouse models of colitis, sodium tungstate (Na_2_WO_4_) administration selectively blunts the expansion of the *Enterobacteriaceae* population, whereas other major taxonomic families remain unchanged (139). These findings illustrate that elucidating the ecological causes of an elevated *Enterobacteriaceae* abundance during colitis (86, 90) directly informs the development of strategies for precision editing of the gut microbiota to normalize the abundance of this taxon during colitis (139).

Notably, when germ-free mice are engrafted with gut microbiota from IBD patients with active flares and inflammation is induced by treatment with DSS, tungstate administration selectively reduces the intestinal *Enterobacteriaceae* load and decreases markers of mucosal inflammation (139). Similarly, in mice with an intact microbiota, tungstate administration reduces the severity of DSS-induced colitis by normalizing the abundance of *Enterobacteriaceae* (139–141). In contrast, DSS-induced inflammation in germ-free mice does not respond to tungstate administration (139), demonstrating that tungstate reduces intestinal inflammation by acting on the microbiota. Thus, precision editing of the gut microbiota identifies a bloom of *Enterobacteriaceae* as a signature of dysbiosis that is causatively linked to an exacerbation of intestinal inflammation during IBD.

### Metabolism-Based Editing of the Microbiota: CRC.

Gut dysbiosis is associated with CRC (142), the third most diagnosed cancer worldwide (143). Only about 20% of CRC cases can be attributed to familial history (144), pointing to environmental factors as important contributors to tumorigenesis. A prime candidate for these environmental factors is the presence of pathobionts in the gut microbiota. For example, colibactin-producing *E. coli* (9), toxin-producing *Bacteroides fragilis* (phylum *Bacteroidota*) (145, 146) and *Fusobacterium nucleatum* (phylum *Fusobacteriota*) (147) can potentiate intestinal tumorigenesis in animal models. These data establish causative links between the gut microbiota and tumorigenesis, but the evidence they provide to connect enhanced tumor formation with an elevated pathobiont abundance during dysbiosis remains correlative.

**Fig. 3.**
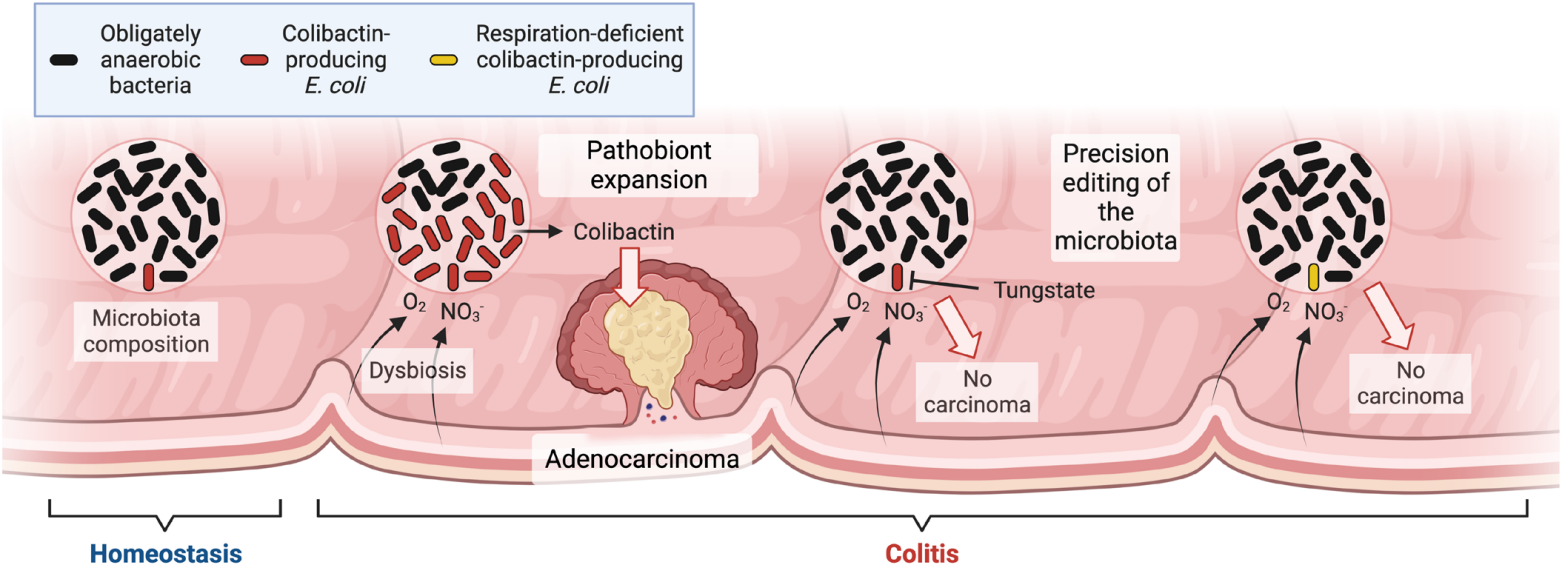
Precision editing of the microbiota causatively links pathobiont expansion during dysbiosis to CRC formation. Colibactin-producing *E. coli* are minority species within the gut microbiota during homeostasis (*Left*). Colitis triggers dysbiosis by increasing the availability of host-derived oxygen (O_2_) and nitrate (NO_3_^−^). Oxygen and nitrate fuel growth of colibactin-producing *E. coli* to promote adenocarcinoma formation (*Center*), whereas blocking a bloom of the pathobiont through precision editing prevents carcinoma (*Right*). Created with BioRender.com.

Colibactin-producing *E. coli* are a good example of a pathobiont with elevated abundance during dysbiosis. Intestinal inflammation is a risk factor for developing CRC (148, 149) and this condition correlates with an increase in the abundance of colibactin-producing *E. coli* in the gut microbiota (9) (Fig. 3). Colibactin is a genotoxin that alkylates DNA on adenine residues (150, 151) and induces double-strand breaks in cultured cells (152). Between 2% and 6.3% of human CRC cases have a mutational signature characteristic of colibactin (153), demonstrating that colibactin-producing *E. coli* have a direct role in the occurrence of oncogenic mutations in patients. Interleukin-10-deficient germ-free mice develop colitis and adenocarcinomas upon azoxymethane (AOM) treatment when they are monoassociated with colibactin-producing *E. coli*, but tumor formation is markedly reduced upon monoassociation with an *E. coli* mutant deficient for colibactin synthesis (9). Collectively, these data show that inflammation correlates with a dysbiotic expansion of a pathobiont with genotoxic potential.

The consequences of a dysbiotic expansion of colibactin-producing *E. coli* on tumor formation can be explored using precision editing of the gut microbiota. The ecological cause for an elevated abundance of *E. coli* in DSS/AOM-treated mice is an increased availability of host derived oxygen and nitrate (93, 123). One approach for precision editing of the microbiota is based on *Enterobacteriaceae-*free mice that are available from some commercial vendors (154). By engrafting littermates of *Enterobacteriaceae-*free mice with either colibactin-producing *E. coli* or *E. coli* mutants lacking pathways required for enhancing their abundance during colitis, it is possible to address the question whether the expansion of a pathobiont in the microbiota exacerbates disease (55). Work in this model shows that DSS/AOM-treated mice develop colitis and adenocarcinomas when they are engrafted with colibactin-producing *E. coli*, but tumor formation is markedly reduced upon engraftment with an aerobic respiration-deficient *E. coli* mutant, which can no longer enhance its abundance during inflammation (93). A second approach for selectively blocking an expansion of colibactin-producing *E. coli* during gut inflammation is tungstate administration, which reduces tumor formation in DSS/AOM-treated mice (123), thus causatively linking pathobiont expansion during dysbiosis to tumorigenesis (Fig. 3).

Collectively, these two approaches for precision editing of the gut microbiota provide complementary evidence that dysbiosis enhances tumorigenesis of the gut microbiota by specifically increasing the abundance of a pathobiont with genotoxic potential (93, 123). Each method for establishing a causative link between changes in the microbiota composition and CRC formation relies on information about the ecological causes of dysbiosis that were discovered through infectious disease research. In turn, the development of methods for precision editing of the microbiota provides a blueprint for investigating the role of dysbiosis in other diseases.

### Changes in Microbial Metabolism during Dysbiosis.

The traditional definition of dysbiosis as a shift in the bacterial species configuration (17) focuses research on establishing links between compositional and functional changes in the gut microbiota. However, the concept that dysbiosis is characterized by an underlying change in host physiology (24, 63, 65, 68) raises the possibility that altering the growth conditions of the gut microbiota could shift microbial metabolism, an aspect of dysbiosis that might be missed by microbiota profiling.

One harmful metabolite exclusively derived from the gut microbiota is trimethylamine (TMA), which is produced during the catabolism of choline or carnitine, two nutrients abundant in red meat (155). Gene clusters encoding enzymes for choline or carnitine catabolism are present in phylogenetically diverse members of the gut microbiota, including representatives of the classes *Gammaproteobacteria*, *Deltaproteobacteria* (phylum *Pseudomonadota*), *Coriobacteriia* (phylum *Actinomycetota*), *Bacilli,* and *Clostridia* (156–158). Microbiota-derived TMA is absorbed by the host and converted by flavin monooxygenases in the liver to the uremic toxin trimethylamine-*N-*oxide (TMAO) (159). Plasma levels of TMAO are elevated in patients with CVD (155, 160, 161), CKD (162) and type 2 diabetes (163). Microbiota-derived TMAO accelerates disease in mouse models of atherosclerosis (155, 161) and CKD (164, 165). Conversely, targeted inhibition of microbiota-derived TMA production using a structural choline analog attenuates atherosclerosis (166) and slows progression of CKD in mouse models (167). Collectively, these data causatively link gut microbiota-derived TMA to leading causes of human morbidity and mortality.

Gut dysbiosis accompanies CVD, CKD, and type 2 diabetes, and in each disease, the fecal microbiota of patients commonly features an increase in the abundance of taxa within the *Gammaproteobacteria*, such as *E. coli* (6, 8, 10, 168). One unanswered question in the field is whether dysbiosis generates a “uremic microbiota” with increased production of metabolites, such as TMA, thereby heightening levels of uremic toxins in the plasma of patients (169). Compositional changes in the microbiota from patients with CKD do not result in increased uremic toxin production during in vitro anaerobic batch culture (170). But a limitation of this approach is that in vitro culture conditions do not resemble the gut environment of patients. Notably, nitrate is required for choline catabolism during in vitro anaerobic batch culture of *E. coli* (124). In a mouse model of high fat intake, dysbiosis features an elevated *E. coli* abundance (171) and a raised availability of host-derived oxygen and nitrate in the colonic lumen (124). Inoculation of *Enterobacteriaceae-*free mice with a TMA-producing *E. coli* strain increases TMAO levels in the serum when animals receive a high-fat diet supplemented with choline but not when animals are fed a low-fat chow supplemented with choline (124). Importantly, TMA production by *Enterobacteriaceae* is driven by host-derived nitrate because treatment with aminoguanidine, a chemical inhibitor of the host enzyme iNOS (172), blocks *E. coli* from increasing the TMAO serum levels in mice receiving high-fat diet supplemented with choline (124). Thus, host-derived nitrate powers choline catabolism of *E. coli* in vivo, a feature of dysbiosis that cannot be recapitulated during in vitro anaerobic batch culture in the absence of nitrate (170).

The emerging picture implies that an underlying change in host physiology (i.e., increased iNOS synthesis) can shift microbial metabolism (i.e., induce nitrate respiration-dependent choline catabolism in *E. coli*) to increase the production of uremic toxins during dysbiosis (124). This example illustrates how insights into the ecological causes of dysbiosis can directly inform approaches to tackle the question whether dysbiosis increases production of harmful metabolites by the gut microbiota.

## Conclusions

At the dawn of modern microbiome research, massive amounts of data were generated when a theoretical framework seemed elusive, which made it challenging to approach questions using hypothesis-driven research (173). However, rather than rejecting hypothesis-driven research altogether (174), we suggest that understanding our microbiome requires supplementing large datasets and sophisticated algorithms to analyze them with basic microbiological concepts.

Studying how enteric pathogens manipulate host functions that control the growth conditions at mucosal surfaces has taught us that one of the ecological causes of dysbiosis in the large intestine is an increased availability of host-derived electron acceptors. This concept is relevant for understanding dysbiosis in many noninfectious human conditions because the underlying principles apply to microbial communities in general. Specifically, the environment selects for those microbes that use the redox reactions generating the greatest free energy, thus making electron acceptors the dominant drivers of microbial community composition in nature (175). Embracing this theoretical framework goes a long way in explaining the longitudinal and cross-sectional heterogeneity of the gut microbiota (176) and understanding the ecological causes of compositional changes during dysbiosis (53, 67).

The finding that changes in the microbiota composition can contribute to chronic human diseases has focused strategies to remediate dysbiosis on microbiota-based therapeutics, such as probiotics, prebiotics, precision editing of the microbiota, or fecal microbiota transplantation (177). However, the underlying cause for alterations in the microbiota composition is a change in the host environment, which represents an important component of the microbiome (22–24). Thus, in addition to targeting the microbes with microbiota-based therapeutics, dysbiosis can be remediated by targeting the organ in which the microbiota resides in with drugs to normalize host functions that control microbial growth on host surfaces (65). This concept provides a starting point for developing therapeutic strategies to remediate dysbiosis by targeting its underlying cause in the host. Exploring this strategy along with microbiota-based therapeutics might provide alternatives to traditional clinical interventions for a broad spectrum of chronic diseases, which makes this line of inquiry one of the most exciting areas in human microbiome research.

## Data Availability

There are no data underlying this work.
